# Impact on Quality of Life of Seton Placing in Perianal Crohn's Disease

**DOI:** 10.3389/fsurg.2021.806497

**Published:** 2022-01-24

**Authors:** Imerio Angriman, Monica Tomassi, Cesare Ruffolo, Giovanni Bordignon, Luca Saadeh, Mario Gruppo, Salvatore Pucciarelli, Romeo Bardini, Marco Scarpa

**Affiliations:** ^1^Chirurgia Generale III, Department of Surgical Oncological and Gastroenterological Sciences, Azienda Ospedale Università di Padova, Padua, Italy; ^2^General Surgery Unit, Azienda Ospedale Università di Padova, Padua, Italy; ^3^Surgical Oncology Unit, Veneto Institute of Oncology IOV Istituto di Ricerca e Cura a Carattere Scientifico (IRCCS), Castelfranco Veneto, Italy

**Keywords:** Crohn's disease, perianal fistula, non-cutting seton, quality of life, surgery

## Abstract

**Introduction:**

Often, in perineal Crohn's disease (CD), a seton is placed to guarantee a constant drainage and prevent septic complication while biologic therapy is ongoing. This study aimed to assess the long-term quality of life after surgery for perineal CD in relation to seton placing.

**Patients and Methods:**

Data of 65 consecutive patients with CD and non-CD operated on from 2014 to 2019 for perianal fistula or abscess were retrieved. Forty-three had CD and 14 of them had a seton placed during surgery and they kept it on while they had anti-TNF-alpha therapy. Patients were interviewed with the Cleveland Global Quality of Life (CGQL) and SF-12 quality of life questionnaires. Disease activity was defined as Harvey-Bradshaw Index (HBI) and Perianal Disease Activity Index (PDAI). Comparisons between groups were carried out with the nonparametric tests, and multiple regression models were used to assess predictors of quality of life.

**Results:**

The total CGQL score and SF-12 mental component score (MCS) were significantly higher (and thus better) in the seton group than in patients treated without seton. On the contrary, SF-12 physical component score (PCS) was not different between the two groups. HBI was significantly better in patients in the seton group. At multivariate analysis, seton placement and HBI were confirmed to be independent predictors of long-term SF-12 MCS whereas only HBI confirmed to be a predictor of total CGQL score.

**Conclusions:**

Seton placing during anti-TNF-alpha therapy is independently associated with a better MCS. Unexpectedly, this device, instead of to cause psychological distress, seems to assure patients during their biologic therapy providing psychological benefit beyond the mere medical effect.

## Introduction

During the natural course of Crohn's disease, up to one-third of patients develop perianal fistula (1). Perianal Crohn's disease is associated with perianal pain, discharge, and permanent local anatomical alterations. It results as disabling conditions characterized by a greatly diminished quality of life, directly affecting patients' physical, psychological, and sexual aspects of life (2, 3). However, despite Crohn's perianal fistulas exerting a heavy negative physical and emotional impact on patients (4), currently, there is almost no specific patient-derived quality-of-life tools to measure response to treatment. The only one, the very recently created questionnaire, the Crohn's Anal Fistula Quality of Life (CAF-QoL), has not been used yet in a center different from the one where it was created (5). There is thus a relative paucity of information about the quality of life in patients with perianal CD.

Management of perianal Crohn's disease is complex: the multimodality approach [surgery and medical therapy] is now accepted as the best treatment option (5, 6). A recent multicenter randomized controlled trial (PISA) compared sole chronic seton vs. anti-TNF treatment vs. combined surgical closure of internal opening following anti-TNF induction therapy in patients with CD with high PF and single internal opening but was terminated early due to unacceptably higher reintervention rates in chronic seton arm (7). Therefore, multimodality treatment with anti-TNF-alpha therapy and placement of non-cutting seton have shown a significant reduction in the risk of repeated surgery, and permanent disability remains the main approach to perianal CD (5).

Thus, surgery is the first step of the multidisciplinary treatment: it assures abscess and/or fistula drainage, minimizing the risk of septic complications (8, 9). Long-term goals of surgical treatment include fistula healing, reducing recurrences, minimizing sphincter damage, avoiding proctectomy, and so improve patients' quality of life (8, 10). In case of simple fistulas, superficial or low intersphincteric ones, open fistulotomy can be performed, with 80–100% healing rate and good functional results (11). Instead, complex fistulas often require placement of non-cutting seton. Non-cutting staged fistulotomy can be temporally used before a definitive surgical repair or, in case of failed definitive surgical repairs or multiple synchronous fistulas, it could be used as long-term treatment preventing abscess formation (12, 13). Seton placing promotes fibrosis avoiding premature skin closure, which guarantees controlled long-term fistula drainage preserving the integrity of external anal sphincter (14, 15). With a reduced risk for septic complications, patients could have their biologic therapy continued, increasing the effectiveness of medical therapy (16).

However, there is still a relative controversy about the quality of life in patients with perianal CD undergoing surgery. Therefore, our study aimed to assess the long-term quality of life after surgery for perineal CD.

## Patients and Methods

### Study Design

This is a prospective monocentric observational study on the quality of life of CD patients with perianal disease. Data of all the consecutive patients, operated for perianal fistula or abscess at the Department of Surgical and Gastroenterological Science of Padova University from 2014 to 2019, were collected. All participants provided informed consent. The study was performed according to the principles of the Declaration of Helsinki, and it was submitted to the Ethical Committee of the Azienda Ospedaliera di Padova (QoLPCD project).

Demographic data, medical and surgical history, and clinical and MRI features of perianal disease were retrieved. Previous surgery for perianal disease was considered, but only the clinical, anatomical, and surgical aspects of the latest intervention were recorded. Fistula was classified according to AGA classification (10). Patients were included if they had at least a six-month follow-up after surgery. Since the study was carried out during the COVID pandemic, all patients were interviewed by oral (telephone) or email questionnaires.

### Questionnaires

Quality of life was assessed with the Cleveland Global Quality of Life (CGQL) and SF-12 quality of life questionnaires. Disease activity was defined as Harvey-Bradshaw Index (HBI) and Perianal Disease Activity Index (PDAI). The CGQL is a 3-item questionnaire created to analyze the generic QoL of patients undergoing restorative proctocolectomy for ulcerative colitis (17). It was translated and validated in the Italian language by our research group and used in the same patients (18) and in patients with CD undergoing ileocolonic resection (19). The SF12 is a 12-item generic questionnaire including a mental component score (MCS) and a physical component score (PCS) deriving from the ampler SF-36 (20). Disease activity was quantified with the Harvey-Bradshaw Activity Index for CD that includes 5 items covering symptoms of patients with CD (21).

### Outcome Measures and Sample Size

Primary outcome measures were general quality of life (CGQL global score) and SF-12 MCS and PCS in patients with CD and in patients with sporadic perianal fistula. Secondary outcome measures were general quality of life (CGQL global score) and SF-12 MCS and PCS in patients with CD who had seton compared with those in patients with CD who had not. Quality-of-life scales were evaluated at least 6 months after surgery. The sample size was calculated setting alpha (type I error) at 0.05 and beta (type 2 error) at 0.20 and an effect size at 1. Therefore, the final sample size was at least 16 patients for each group.

### Surgical Management

The most common indication for operative intervention with perianal CD was septic complications, which include abscesses or undrained fistulas (22). In case of symptomatic simple fistula, seton placement or fistulotomy was used. Setons were used to keep fistulous tracts open, allowing them to drain to prevent reaccumulation of undrained sepsis (23). Also, setons were left in place long-term whereas medical treatments are used (24). Moreover, an endorectal mucosal advancement flap was a procedure that we used to close the internal fistula opening, usually in case of rectal vaginal fistula (25). Finally, fecal diversion with a stoma formation was used to divert the fecal stream away from wounds to allow healing in case of extremely complex perianal disease.

### Patients' Selection

**Figure 1:**
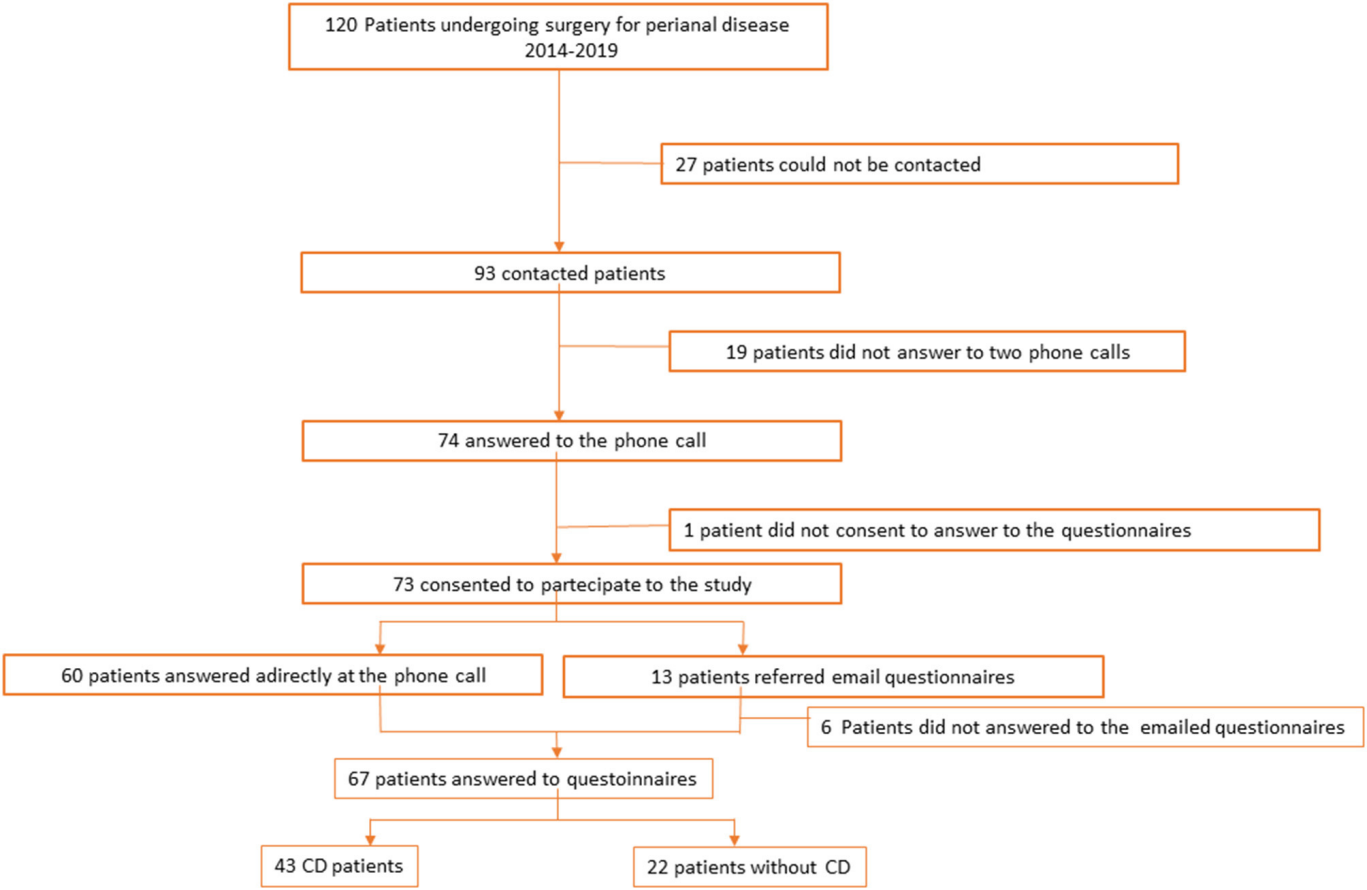
Patients selection.

Data of 120 consecutive patients, operated for perianal fistula or abscess at the Department of Surgical and Gastroenterological Science of Padova University from 2014 to 2019, were collected. In total, 46 patients were lost at the follow-up, and 74 were contacted by phone call and asked to participate but only 65 actually completed the survey and 43 of them had CD. In this group, 14 patients of them had a seton placed during surgery and they kept it on while they had anti-TNF-alpha therapy. Some patients presented at diagnosis with more than one fistula. Patients' selection is shown in Figure 1.

### Patients' Characteristics

In the group of patients with CD, there were 17 women and 26 men and their median age at the onset of perianal disease was 37 (15–75) years. Their median age at CD diagnosis was 27.5 (8–63) years. In this group, 31 patients had previous interventions for perianal disease. The extent and the localization of the perianal disease were not different in patients with CD and in sporadic patients. Similarly, type of surgical intervention was not different in the two groups. Only ostomy creation tended to be more frequent in patients with CD. On the contrary, patients with CD were more frequently having chronic medical therapy. Patients' characteristics are shown in Table 1.

**Table 1:** Demographic data, anatomical description, AGA classification, and clinical history of 66 patients who completed the survey.

	**Sporadic**	**22**	**Crohn**	**43**	***P*** **value**
	***N***	**%**	***N***	**%**	
**Gender**					
Female/Male	1	5	4	9	0.65
**Age**					
Median age at onset of perianal disease (years)	37 (15–75)				
Median age at CD diagnosis (years)			27.5 (8–63)	
**Perianal disease**					
Perianal abscess	1	5	4	9	0.65
Complex abscess	8	36	17	40	0.99
**Simple**					
Low intersphincteric fistula	4	18	10	23	0.75
Submucosal fistula	2	9	4	9	0.99
**Complex**					
Ano-vulvar or ano-vaginal fistula	0	0	5	12	0.15
Transsphincteric fistula	5	23	8	19	0.74
High intersphincteric fistula	3	14	4	9	0.68
Supersphinteric fistula	0	0	2	5	0.54
**Therapy**					
Previous intervention for perianal disease yes/no	10	45	31	72	0.05
Current chronic medical therapy yes/no biologic therapy yes/no	23	914	2,827	6,563	0.01
**Surgery**					
Associated major surgery (laparotomy, muscle transposition) current medical therapy yes/no	12	59	228	565	0.99
**Surgery**					
Incision, toilette, and drainage associated major surgery (laparotomy, muscle transposition)	131	595	232	545	0.79
Fistulectomy incision, toilette, and drainage	613	2,759	1,723	4,054	0.41
Fistulotomy–fistulectomy	96	4,127	1,317	3,040	0.41
Mucosal flap fistulotomy	29	941	213	530	0.59
Seton mucosal flap	62	279	142	335	0.77
Seton present at the study enrollment seton	16	527	214	533	0.99
Ostomy seton present at the study enrollment	01	05	62	145	0.08
Ostomy	0	0	6	14	0.08

### Statistical Analysis

Statistical analysis was performed using Origin 8.1 software (Origin Lab, Northampton, Massachusetts, USA). Continuous data were expressed as median (IQR) whereas HRQL scores were expressed as mean and standard error (SE). Categorical data is expressed as number and percentage (%). The nonparametric Mann–Whitney *U* test was used to compare groups, whereas multiple regression models were used to assess predictors of quality of life. All tests were two-sided and a *p* < 0.05 was considered statistically significant.

## Results

### Impact of Perianal Disease on the Quality of Life

**Figure 2:**
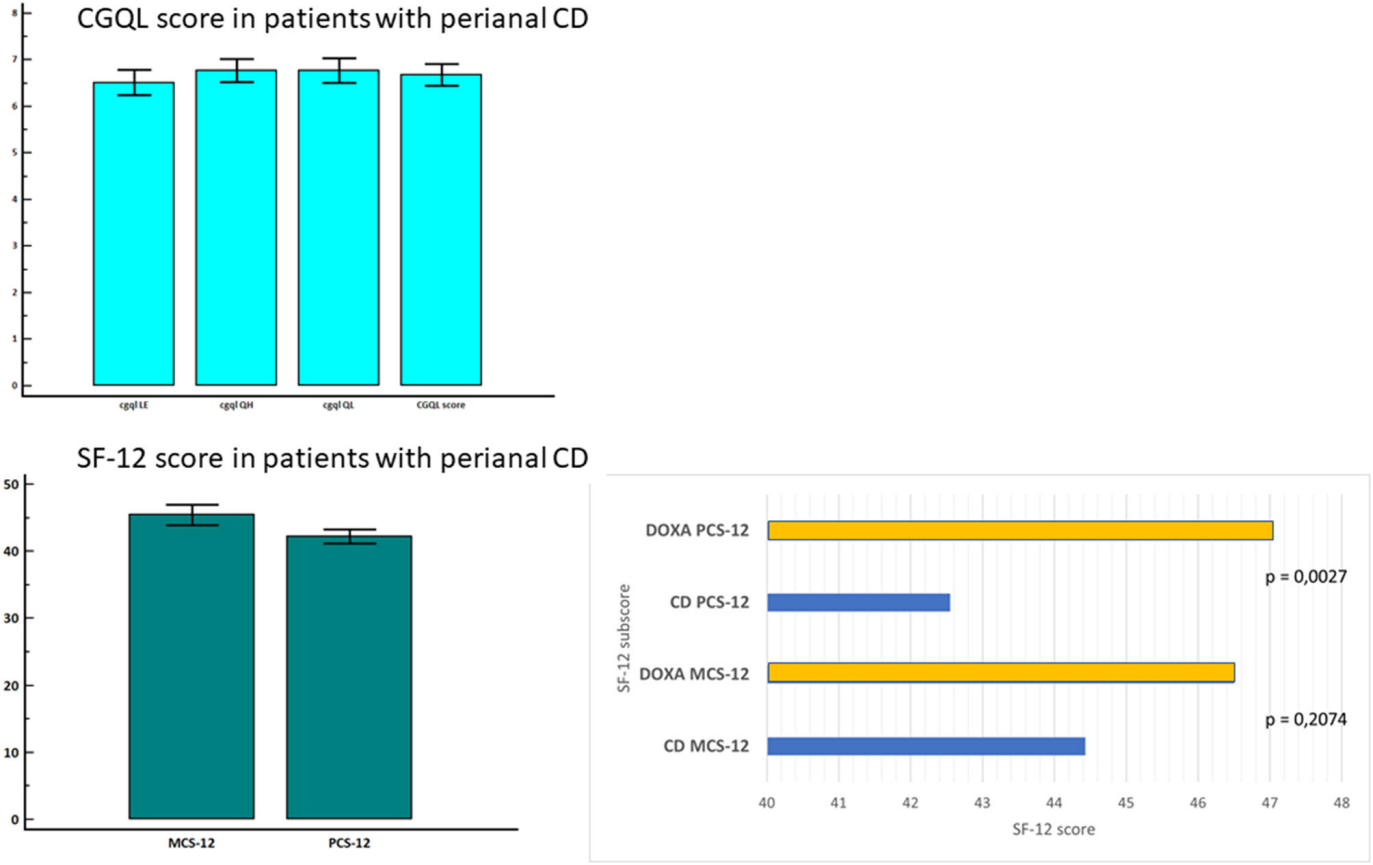
Quality of life in patients with CD with perianal disease and in healthy controls.

Patients with perianal CD had a low global quality of life, current level of energy, current quality of life, and current quality of health as measured with CGQL. The comparison with the Italian norms, as measured with SF-12 (26), showed that patients with CD with perianal disease had a similar MCS but a significantly lower PCS (*p* = 0.002). The impact of perianal disease on the quality of life of patients with CD is shown in Figure 2.

**Figure 3:**
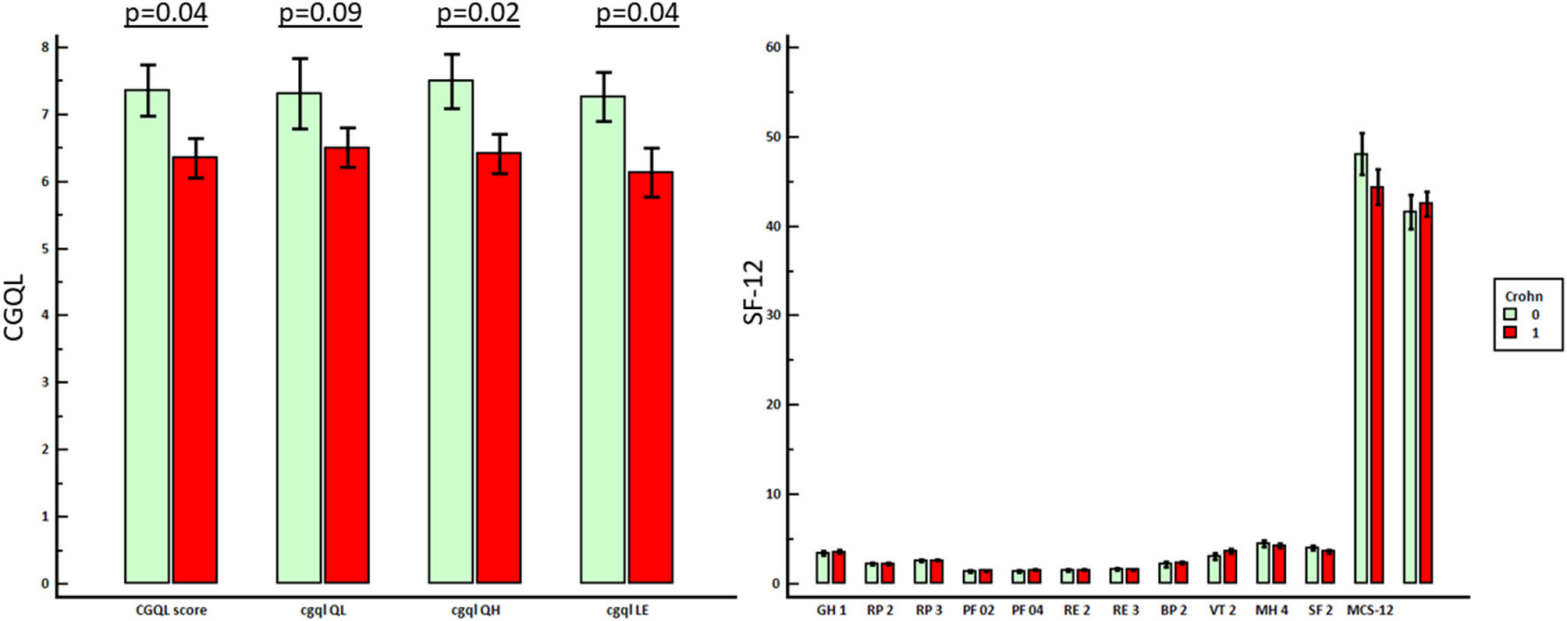
Comparison between the quality of life of patients with perianal CD and patients with sporadic perianal disease.

All the scores for global quality of life, current level of energy, current quality of life, and current quality of health, as measured with CGQL, were significantly lower in patients with CD compared to that of patients with sporadic perianal disease. On the contrary, either MCS or PCS was similar in patients with CD compared to sporadic perianal disease patients. The comparison of quality of life in CD and sporadic perianal disease is shown in Figure 3.

### Impact of Seton Placing on Quality of Life

Seton was placed in 14 patients with perianal CD. As shown in Table 2, these patients had more frequently a complex perianal disease, and more often, they were on biologic therapy, such as infliximab or adalimumab than those who had not a seton placed (*p* = 0.006 and *p* = 0.04, respectively). Moreover, patients who had a seton placed had more frequently undergone to fistulectomy whereas patients without seton had more often undergone incision, toilette and drainage, and/or fistulotomy (*p* = 0.0001, *p* = 0.007, and *p* = 0.03, respectively).

**Table 2:** Demographic data, anatomical description, AGA classification, and clinical history of the 43 patients with perianal CD who completed the survey.

	**Seton**	**14**	**No seton**	**29**	***P*** **value**
	***N***	**%**	***N***	**%**	
**Perianal disease**					
Perianal abscess	0	0	4	14	0.28
Complex abscess	10	71	7	24	0.006
**Simple**					
Low intersphincteric fistula	5	36	5	17	0.25
Submucosal fistula	0	0	4	14	0.28
**Complex**					
Ano-vulvar or ano-vaginal fistula	3	21	2	7	0.30
Transsphincteric fistula	3	21	5	17	0.70
High intersphincteric fistula	1	7	3	10	0.99
Supersphinteric fistula	1	7	1	3	0.99
**Therapy**					
Previous intervention for perianal disease yes/no	12	86	19	66	0.27
Biologic therapy yes/no	12	86	15	52	0.04
Current medical therapy yes/no	11	79	17	59	0.30
**Surgery**					
Associated major surgery (laparotomy, muscle transposition)	0	0	2	7	0.99
Incision, toilette, and drainage	3	21	20	69	0.007
Fistulectomy	13	93	4	14	0.0001
Fistulotomy	1	7	12	41	0.03
Mucosal flap	0	0	2	7	0.99
Ostomy	2	14	4	14	0.99

**Figure 4:**
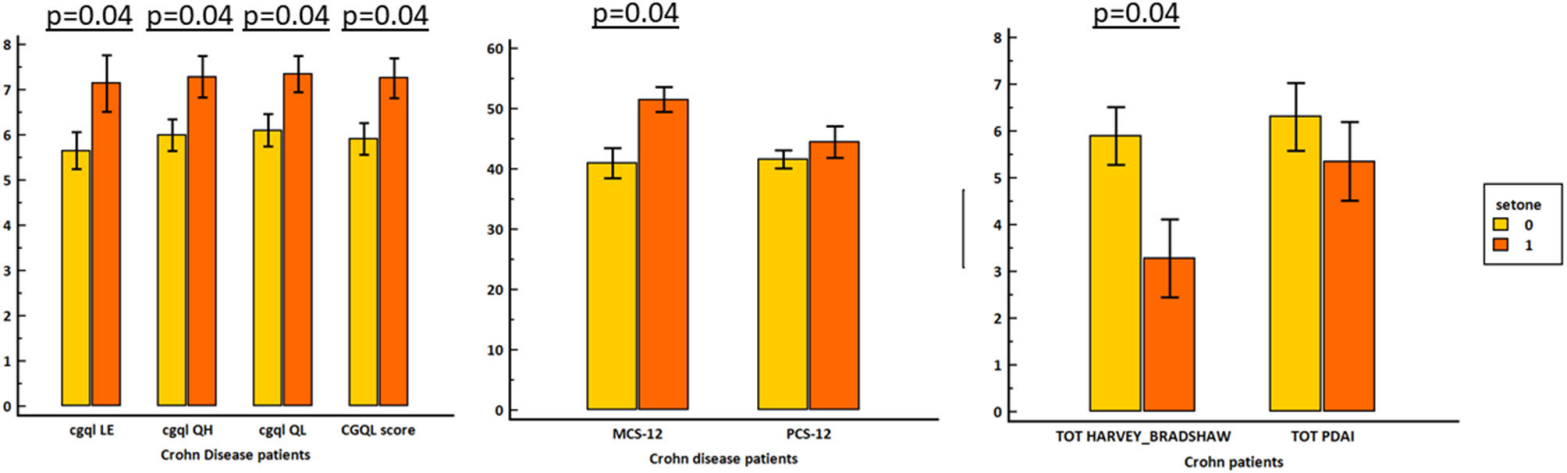
Effect of seton placement on quality of life and disease activity.

The total CGQL score and SF-12 MCS were significantly higher (and thus better) in the seton group than in patients treated without seton (*p* = 0.03 and *p* = 0.02, respectively). On the contrary, SF-12 PCS was not different between the two groups. HBI was significantly lower in patients in the seton group (*p* = 0.01). As shown in Table 3, at multivariate analysis, seton placement and HBI confirmed to be independent predictors of long-term SF-12 MCS whereas only HBI confirmed to be a predictor of total CGQL score. The impact of seton placing on quality of life is shown in Figure 4.

**Table 3:** Predictors of MCS component of SF12.

**Independent variables**	**Univariable analysis**	**Multivariable analysis**	
	**Correlation r**	**Multiple regression coefficient**	***P*** **value**
HBI	−0.345	−7.86381	0.0083
Seton placing	0.325	7.79678	0.0142

## Discussion

Perianal CD is associated with perianal pain, discharge, and permanent local anatomical alterations that directly affect patients' physical, psychological, and sexual aspects of life (2, 5). Often, a seton is placed to provide a continuous drainage during anti-TNF-alpha therapy to prevent septic complications, and the constant presence of this device might somehow influence the perception of the quality of life of these patients. However, despite CD perianal disease has a heavy negative physical and emotional burden on patients (3), currently, there are relatively few studies assessing quality of life of these patients and even less specifically in relation to a seton placing. There is only a disease-specific questionnaire specifically dedicated to this condition, the CAF-QoL (4), and this is a sort of index of the relative paucity of information about quality of life in patients with perianal CD. Therefore, our study aimed to assess the long-term quality of life after surgery for perineal CD in relation to a seton placing.

In our series, patients with perianal CD had a low global quality of life, current level of energy, current quality of life, and current quality of health as measured with CGQL, and the comparison with the Italian norms (26) showed that patients with CD with perianal disease had a similar SF-12 MCS but a significantly lower SF-12 PCS. A recent Italian study showed that anorectal function is impaired in patients with IBD with perianal disease, and this impairment, such as fecal incontinence, correlates with poorer quality of life (27). A recent systematic review pointed out that up to 59% of patients with CD with complex perianal fistulas are at the risk of fecal incontinence and thus of greatly diminished quality of life (28). These data, taken together, suggest that the CD perianal disease causes a deep impairment of quality of life due to the physical symptoms.

In our series, global quality of life, current level of energy, current quality of life, and current quality of health, as measured with CGQL, were significantly lower in patients with CD compared to that of patients with sporadic perianal disease. In a recent British study, low utility values were assigned to the non-remission health states for perianal fistulae in CD by the general public and patients with CD suggesting the high burden of inadequately managed perianal fistula in CD (29). In our opinion, all these data demonstrate that is the condition of permanence, of never healing, that makes this condition so difficult to bear and the burden on quality of life so heavy.

In our series, the total CGQL score and SF-12 MCS were significantly better in the seton group than in patients treated without seton. On the contrary, SF-12 PCS was not different between the two groups. HBI was significantly lower in patients in the seton group. At multivariate analysis, seton placement and HBI confirmed to be independent predictors of long-term SF-12 MCS. Similarly, in an Austrian study, multiple variable analysis showed that type of operation, more than one perianal fistula opening, and active Crohn's disease were independent risk factors for a worse quality of life (5). recent PISA randomized controlled trial that compared the chronic seton quality of life was not impaired compared to anti-TNF treatment or combined surgical closure of internal opening following anti-TNF induction therapy (7). In a very recent study, no significant differences were found on Wexner scale, CGQL, IBDQ, and SF-36 between non-cutting seton and fistulotomy group (30). In our opinion, not only this device did not cause any quality-of-life impairment on itself but also, unexpectedly, instead to cause a psychological distress, seems to assure patients during their biologic therapy providing psychological benefit beyond the mere medical effect.

In conclusion, our study showed that the CD perianal disease causes a deep impairment of quality of life due to the physical symptoms and, that is the condition of permanence, of never healing, that makes this condition so difficult to bear and the burden on quality of life so heavy. However, not only non-cutting seton placement did not cause any quality-of-life impairment on itself but also, unexpectedly, instead to cause a psychological distress, seems to assure patients during their biologic therapy providing psychological benefit beyond the mere medical effect.

## Data Availability Statement

The raw data supporting the conclusions of this article will be made available by the authors, without undue reservation.

## Ethics Statement

The studies involving human participants were reviewed and approved by Comitato Etico Scientifico dell'Azienda Ospedale Università di Padova. The patients/participants provided their written informed consent to participate in this study.

## Author Contributions

IA, CR, SP, RB, and MS conceived and designed the study. MT, GB, LS, and MG interviewed patients and collected data. IA, CR, and MS analyzed the data and drafted the paper. MT, GB, LS, MG, SP, and RB revised the paper for important intellectual content. All authors have read and approved the final version of the paper.

## Funding

This study was partially funded by SID 2019 grant on Fibrosis in Crohn's Disease from the University of Padova to IA.

## Conflict of Interest

The authors declare that the research was conducted in the absence of any commercial or financial relationships that could be construed as a potential conflict of interest. The handling editor declared a past co-authorship with several of the authors IA, SP, and MS.

## Publisher's Note

All claims expressed in this article are solely those of the authors and do not necessarily represent those of their affiliated organizations, or those of the publisher, the editors and the reviewers. Any product that may be evaluated in this article, or claim that may be made by its manufacturer, is not guaranteed or endorsed by the publisher.

## References

[1] Schwartz DA Loftus Jr EV Tremaine WJ Panaccione R Scott Harmsen W Zinsmeister AR. The natural history of fistulizing Crohn's disease in Olmsted County, Minnesota. Gastroenterology. (2002) 122:875–80. 10.1053/gast.2002.32362 11910338

[2] Mahadev S Young JM Selby W Solomon MJ. Quality of life in perianal Crohn's disease: what do patients consider important? Dis Colon Rectum. (2011) 54:579–85. 10.1007/DCR.0b013e3182099d9e 21471759

[3] Adegbola SO Dibley L Sahnan K Wade T Verjee A Sawyer R. Burden of disease and adaptation to life in patients with Crohn's perianal fistula: a qualitative exploration. Health Qual Life Outcomes. (2020) 18:370. 10.1186/s12955-020-01622-7 33218361 PMC7678264

[4] Adegbola SO Dibley L Sahnan K Wade T Verjee A Sawyer R. Development and initial psychometric validation of a patient-reported outcome measure for Crohn's perianal fistula: the Crohn's Anal Fistula Quality of Life (CAF-QoL) scale. Gut. (2021) 70:1649–56. 10.1136/gutjnl-2019-320553 33272978 PMC8355881

[5] Riss S Schwameis K Mittlböck M Pones M Vogelsang H Reinisch W. Sexual function and quality of life after surgical treatment for anal fistulas in Crohn's disease. Tech Coloproctol. (2013) 17:89–94. 22956209 10.1007/s10151-012-0890-x

[6] Sebastian S Black C Pugliese D Armuzzi A Sahnan K Elkady SM. The role of multimodal treatment in Crohn's disease patients with perianal fistula: a multicentre retrospective cohort study. Aliment Pharmacol Ther. (2018) 48:941–50. 10.1111/apt.14969 30226271

[7] Wasmann KA de Groof EJ Stellingwerf ME D'Haens GR Ponsioen CY Gecse KB. Treatment of perianal fistulas in Crohn's disease, seton vs. anti-TNF versus surgical closure following anti-TNF [PISA]: a randomised controlled trial. J Crohns Colitis. (2020) 14:1049–56. 10.1093/ecco-jcc/jjaa004 31919501 PMC7476637

[8] Gecse KB Bemelman W Kamm MA Stoker J Khanna R Ng SC. IOfIBDIESoC; Robarts Clinical. A global consensus on the classification, diagnosis and multidisciplinary treatment of perianal fistulising Crohn's disease. Gut. (2014) 63:1381–92. 24951257 10.1136/gutjnl-2013-306709

[9] Marzo M Felice C Pugliese D Andrisani G Mocci G Armuzzi G. Management of perianal fistulas in Crohn's disease: an up- to-date review. World J Gastroenterol. (2015) 21:1394–403. 10.3748/wjg.v21.i5.1394 25663759 PMC4316082

[10] Hyder SA Travis SP Jewell DP Mc CMNJ George BD. Fistulating anal Crohn's disease: results of combined surgical and infliximab treatment. Dis Colon Rectum. (2006) 49:1837–1841. 10.1007/s10350-006-0656-5 17041753

[11] Gecse KB Sebastian S Hertogh GD Yassin NA Kotze PG Reinisch W. Results of the fifth scientific workshop of the ecco[ii]: Clinical aspects of perianal fistulising Crohn's disease-the unmet needs. J Crohn's Colitis. (2016) 10:758–65. 10.1093/ecco-jcc/jjw039 26826183

[12] Sandborn WJ Fazio VW Feagan BG Hanauer SB. AGA technical review on perianal Crohn's disease. Gastroenterology. (2003) 125:1508–30. 10.1016/j.gastro.2003.08.025 14598268

[13] Bubbers EJ Cologne KG. Management of complex anal fistulas. Clin Colon Rectal Surg. (2016) 29:43–9. 10.1055/s-0035-1570392 26929751 PMC4755767

[14] Haennig A Staumont G Lepage B Faure P Alric L Buscail L. The results of seton drainage combined with anti-TNFα therapy for anal fistula in Crohn's disease. Colorectal Dis. (2015) 17:311–9. 10.1111/codi.12851 25425534

[15] Buchanan GN Owen HA Torkington J Lunniss PJ Nicholls RJ Cohen CRG. Long-term outcome following loose-seton technique for external sphincter preservation in complex anal fistula. Br J Surg. (2004) 91:476–80. 10.1002/bjs.4466 15048751

[16] Parks AG Stitz RW. The treatment of high fistula-in-ano. Dis Colon Rectum. (1976) 19:487–99. 964106 10.1007/BF02590941

[17] Fazio VW O'Riordain MG Lavery IC Church JM Lau P Strong SA. Long-term functional outcome and quality of life after stapled restorative proctocolectomy. Ann Surg. (1999) 230:575–84; discussion 584–6. 10.1097/00000658-199910000-00013 10522727 PMC1420906

[18] Scarpa M Ruffolo C Polese L Martin A D'Incà R Sturniolo GC. Quality of life after restorative proctocolectomy for ulcerative colitis: different questionnaires lead to different interpretations. Arch Surg. (2007) 142:158–65. 10.1001/archsurg.142.2.158 17309967

[19] Scarpa M Ruffolo C D'Incà R Filosa T Bertin E Ferraro S. Health-related quality of life after ileocolonic resection for Crohn's disease: long-term results. Inflamm Bowel Dis. (2007) 13:462–9. 10.1002/ibd.20080 17206691

[20] Ware J Jr Kosinski M Keller SD. A 12-Item Short-Form Health Survey: construction of scales and preliminary tests of reliability and validity. Med Care. (1996) 34:220–33. 10.1097/00005650-199603000-00003 8628042

[21] Harvey RF Bradshaw JM. A simple index of Crohn's-disease activity. Lancet. (1980) 1:514. 10.1016/s0140-6736(80)92767-1 6102236

[22] Fichera A Michelassi F. Surgical treatment of Crohn's disease. J Gastrointest Surg. (2007) 11:791–803. 10.1007/s11605-006-0068-9 17562122

[23] White RA Eisenstat TE Rubin RJ Salvati EP. Seton management of complex anorectal fistulas in patients with Crohn's disease. Dis Colon Rectum. (1990) 33:587–9. 1694476 10.1007/BF02052212

[24] Geltzeiler CB Wieghard N Tsikitis VL. Recent developments in the surgical management of perianal fistula for Crohn's disease. Ann Gastroenterol. (2014) 27:320–30. 25331917 PMC4188928

[25] Ruffolo C Scarpa M Bassi N Angriman I. A systematic review on advancement flaps for rectovaginal fistula in Crohn's disease: transrectal vs transvaginal approach. Colorectal Dis. (2010) 12:1183–91. 10.1111/j.1463-1318.2009.02029.x 19674019

[26] Kodraliu G Mosconi P Groth N Carmosino G Perilli A Gianicolo EA. Subjective health status assessment: evaluation of the Italian version of the SF-12 Health Survey. Results from the MiOS Project. J Epidemiol Biostat. (2001) 6:305–16. 10.1080/135952201317080715 11437095

[27] Litta F Scaldaferri F Parello A De Simone V Gasbarrini A Ratto C. Anorectal function and quality of life in IBD Patients with a perianal complaint. J Invest Surg. (2021) 34:547–53. 10.1080/08941939.2019.1658830 31625422

[28] Panes J Reinisch W Rupniewska E Khan S Forns J Khalid JM. Burden and outcomes for complex perianal fistulas in Crohn's disease: systematic review. World J Gastroenterol. (2018) 24:4821–34. 10.3748/wjg.v24.i42.4821 30479468 PMC6235801

[29] Longworth L Fountain D Singh J Azzabi I Owen G Lundstam U. Elicitation of health-related utility in perianal fistula in Crohn's disease. Patient. (2019) 12:339–48. 10.1007/s40271-018-0352-2 30556095

[30] Gklavas A Sotirova I Karageorgou M Kozonis T Poulaki A Papaconstantinou I. Is the quality of life of patients with fistulizing perianal Crohn's disease impaired by the presence of chronic loose, non-cutting seton? J Gastrointest Surg. (2021) 25:2686–9. 10.1007/s11605-021-04987-2 33772403

